# Oral Microbiome Dynamics in Patients with Acute Lymphoblastic Leukemia and Oral Mucositis

**DOI:** 10.3390/microorganisms14010185

**Published:** 2026-01-14

**Authors:** Ana Elizabeth Sánchez-Becerra, Marcela Peña-Rodríguez, Alejandra Natali Vega-Magaña, Samuel García-Arellano, Hugo Antonio Romo-Rubio, Sony Flores-Navarro, Griselda Escobedo-Melendez, Saray Aranda-Romo, José Sergio Zepeda-Nuño

**Affiliations:** 1 Departamento de Microbiología y Patología, Centro Universitario de Ciencias de la Salud (CUCS), Universidad de Guadalajara, Guadalajara 44340, Jalisco, Mexico; elizabeth.sanchez@academicos.udg.mx; 2 Laboratorio de Diagnóstico de Enfermedades Emergentes y Reemergentes (LaDEER), Centro Universitario de Ciencias de la Salud (CUCS), Universidad de Guadalajara, Guadalajara 44340, Jalisco, Mexico; marce.pena@cucs.udg.mx; 3 Instituto de Investigación en Ciencias Biomédicas (IICB), Centro Universitario de Ciencias de la Salud (CUCS), Universidad de Guadalajara, Guadalajara 44340, Jalisco, Mexico; alejandra.vega@academicos.udg.mx (A.N.V.-M.); samuel.garcia4566@academicos.udg.mx (S.G.-A.); 4 Servicio de Hemato-Oncología Pediátrica, Hospital Civil de Guadalajara “Juan I. Menchaca”, Guadalajara 44670, Jalisco, Mexico; hugoromo48@gmail.com (H.A.R.-R.); gescobedo@hcg.gob.mx (G.E.-M.); 5 Odontología en Servicio de Hemato-Oncología Pediátrica, Hospital Civil de Guadalajara “Juan I. Menchaca”, Guadalajara 44670, Jalisco, Mexico; sflores@gmail.com; 6 Clínica de Diagnóstico, Facultad de Estomatología, Universidad Autónoma de San Luis Potosí, San Luis Potosí 78000, San Luis Potosí, Mexico

**Keywords:** oral microbiome, acute lymphoblastic leukemia, chemotherapy, pediatric oral mucositis

## Abstract

The oral microbiome of patients with acute lymphoblastic leukemia (ALL) undergoes changes caused by the neoplasia as well as the antimicrobial activity of chemotherapy (CTX), which promotes the development of oral mucositis (OM). This study aimed to analyze the oral microbiome dynamics and salivary cytokine production in pediatric ALL patients before and during CTX, comparing children who did and did not develop OM. We conducted a longitudinal, observational, and analytical study including 32 newly diagnosed pediatric ALL patients (ages 2–16 years) undergoing CTX. Oral rinse and non-stimulated saliva samples were collected at baseline (day 0), day 14, and day 21 of induction of CTX, with an additional sample taken during OM episodes when possible. Microbiome analysis was performed using 16S rRNA sequencing on an Illumina MiSeq platform, and salivary cytokines were measured using a Luminex multiplex assay. The most pronounced microbiome changes occurred on day 14, particularly in patients who developed OM, characterized by higher α diversity, increased abundance of opportunistic taxa, and elevated IL-6 concentrations. In contrast, patients who did not develop OM exhibited a more stable microbial composition. Overall, these findings indicate that temporal oral dysbiosis and increased IL-6 may serve as early markers and potential predictors of OM development during chemotherapy in pediatric ALL patients.

## 1. Introduction

The most common malignant neoplasm in children is acute lymphoblastic leukemia (ALL). Despite improvements in its management, this disease has a high incidence and mortality [1]. Chemotherapy (CTX) is the first-line treatment for ALL in pediatric patients [2]. However, CTX induces a strong inflammatory response that increases the risk of developing one of the most common adverse effects related to cancer treatment: oral mucositis (OM) [2,3]. OM occurs in approximately 75% of patients undergoing antineoplastic treatment [2,3]. Due to the development of ulcers with consequent loss of integrity in the oral mucosa, this adverse effect has an additional impact on the homeostasis of the microbiome, leading to dysbiosis [3,4].

CTX induces DNA damage and apoptosis in mucosal and submucosal cells, specifically keratinocytes, fibroblasts and endothelial cells [2,3]. Damaged cells release molecules that have the ability to alter local tissue response through binding to specific receptors that play an important role in initiating inflammation and toxicity. These molecules include free radicals, which are responsible for the upregulation of the NF-kB pathway, which results in the excessive production of inflammatory mediators such as IL-1β, IL-6, and TNF-α, among others [5].

The oral microbiome is one of the most diverse and abundant in the human body. Healthy oral microbiota maintains the symbiotic relationship with the host based on mutualistic benefits, including the activation of cytoprotective pathways in epithelial cells, the counteraction of reactive oxygen species, the displacement of pathogenic bacteria, and the promotion of the integrity of the oral mucosa [6]. There are factors that influence the stability of the microbiome, leading to an imbalance or dysbiosis that causes the transition of a commensal or opportunistic microorganism to a pathogen [7]. It has been reported that the composition of the oral microbiome of patients with ALL undergoes changes before starting CTX; these changes are caused by the neoplasia itself, as well as by the antimicrobial activity and myelosuppression inherent to antineoplastic treatment [3]. As described above, CTX promotes damage to the mucosal barrier, leads to disruption of the microbiome, and allows the growth of pathobionts that negatively impact the ability of oral tissues to remain intact during antineoplastic treatment [3,6,8].

We hypothesize that CTX treatment in pediatric patients with ALL significantly alters the composition of the oral microbiome and the levels of salivary cytokines, and that these changes are associated with the development of oral mucositis (OM). The objective of this study was to analyze the oral microbiome in pediatric patients with ALL before and during CTX treatment and to show the dynamics of the oral microbiome during CTX. Likewise, we aimed to determine the production of cytokines in the saliva of these patients. Oral microbiome characteristics and cytokine concentrations were also compared in patients who did and did not develop OM.

## 2. Materials and Methods

### 2.1. Study Population

The sample included pediatric patients with acute lymphoblastic leukemia (ALL) initiating treatment at Hospital Civil de Guadalajara (approximately 58 per year). Based on the standard formula for sample size calculation in health research, the minimum sample required was 31 patients.

A total of 32 newly diagnosed pediatric ALL patients under CTX treatment (Total therapy XV: prednisone, vincristine, daunorubicin, L-asparaginase, cyclophosphamide, cytarabine, 6-mercaptopurine, imatinib, methotrexate and etoposide) [9], between 2 and 16 years old were recruited from the Pediatric Hemato-Oncology Service of the Hospital Civil de Guadalajara “Juan I. Menchaca”, from November 2019 to November 2021. Patients with active severe systemic infectious processes that are isolated, patients sedated, intubated, hemodynamically unstable, or in the pediatric intensive care unit, patients with metabolic diseases, heart disease, or autoimmune diseases were not included.

### 2.2. Oral Examination and Grading of OM

Oral hygiene was evaluated using the Simplified Oral Hygiene Index (OHI-S) [10]. The presence and degree of caries were recorded based on The International Caries Detection and Assessment System (ICDAS) [11]. Furthermore, the cariogenic risk of the diet was assessed. The oral mucosa was examined to record the occurrence of mucositis and graded according to the World Health Organization classification [12].

The World Health Organization (WHO) distinguishes five grades of oral mucositis severity, ranging from 0 to 4. The scale combines both objective and subjective criteria as follows:

Grade 0: No mucositis;
Grade 1: Soreness/erythema;
Grade 2: Erythema and ulcers; able to tolerate solid food;
Grade 3: Unable to tolerate solid food, but able to tolerate liquids;
Grade 4: Unable to tolerate solids or liquids; oral alimentation is not possible.

This system is universal, easily accessible, and widely used by medical personnel within hospital settings.

**Figure 1:**
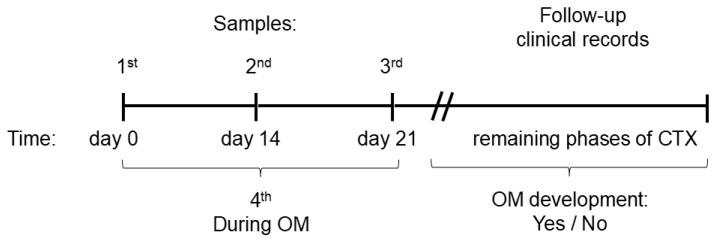
Samples were collected on day 0 (first sample), day 14 (second sample), and day 21 of chemotherapy (third sample). A sample was also collected during the mucositis phase (fourth sample), although only four children presented with symptoms during the sample collection period. Patient follow-up was subsequently reviewed in clinical records to determine how many patients developed mucositis during the remaining phases of chemotherapy. OM: during oral mucositis; No: patients who did not develop oral mucositis; Yes: patients who developed oral mucositis.

Since a few patients developed OM during the sampling period (4 patients), the records were reviewed to determine the total number of children who experienced OM during the remainder of the phases of CTX: induction, consolidation, and maintenance (range: 10 days to 1 year later) (Figure 1).

### 2.3. Oral Sample Collection

The samples were collected during the induction phase of CTX at three time points on days 0, 14, and 21. A fourth sample was taken at the time of the OM episode in those patients who developed it during the sampling period (21 days) (Figure 1). Non-stimulated saliva and oral rinse samples (performed using 3 mL of phosphate-buffered saline solution <PBS> during 30 to 60 s) were collected between 8:00 and 10:00 am in sterile conical tubes. 1 µL/mL of protease and phosphatase inhibitor (MS-SAFE, Sigma Aldrich; catalog number MSSAFE-1VL, St. Louis, MO, USA) was added to the sample. The samples were immediately stored at −80 °C until further analysis.

### 2.4. Microbiome Analyses—DNA Extraction, 16S rRNA PCR Amplification and Sequencing

Bacteria were pelleted from oral rinse samples by centrifugation (SIGMA 1-14K, Osterode am Harz, Germany) at 14,462× *g* for 10 min. DNA was extracted from oral rinse samples using the DNA extraction High Pure PCR Template Preparation Kit (ROCHE, Basel, Switzerland) according to the manufacturer’s instructions. DNA concentration and purity were determined using a Nano-Drop Spectrophotometer (Thermo-Fisher Scientific, Wilmington, DE, USA). Size and integrity of DNA were checked by 1% (*w*/*v*) agarose gel electrophoresis in 0.05‰ (*v*/*v*) GoldView. The extracted DNA was then stored at −20 °C before further analysis.

The amplification of the hypervariable *V3* region of the bacterial *16S* gene was performed using an Illumina Miseq platform (Illumina, Inc., San Diego, CA, USA). All samples were processed and sequenced together. Amplification primers contained adapters for MiSeq sequencing and single-index barcodes, resulting in PCR products that were pooled and sequenced directly.

### 2.5. Bioinformatic Analysis

Libraries (V3 16S) were constructed following the workflow of the 16S Metagenomic Sequencing Library Preparation from Illumina (San Diego, CA, USA). Raw sequencing reads were analyzed on the QIIME 2 2023.2 pipeline [13]. The denoise was performed with DADA2 (version 1.28.0) using a quality score of Q20, truncated forward reads in 265 nt, reverse reads in 220 nt, and employed a depth of 11,200 reads per sample. Subsequently, sequences were classified in Amplicon Variant Sequence (ASVs) using the Silva rRNA Database Project (version 138.1) [14]. Community richness Shannon and Chao1 diversity indices were used to determine α diversity. Bray–Curtis distance and PERMANOVA analysis were applied to β diversity.

The microbiome analyst platform was used for differential abundance analysis. Qiime 2 2023.2 was used to compare the relative abundance (RA) of each taxon at different taxonomic levels between groups. Graphics and statistical analysis were performed with the microbiome analyst platform and GraphPad Prism version 8.

### 2.6. Detection of Interleukins

The saliva samples were used for this purpose. Saliva IL-1β, IL-4, IL-6, IL-8, IL-17, TNF-α and MMP-1 levels were assessed with the Human Magnetic Luminex Assay, premixed multiplex kit (R&D systems; catalog number LXSAHM-07, R&D Systems, Inc., Minneapolis, MN, USA) according to the manufacturer’s instructions. The reading of the samples was carried out in the Magpix multiplex reader (Luminex^®^ MAGPIX^®^, Bio-Rad Bio-Plex analyzer, Luminex Corporation, Austin, TX, USA).

### 2.7. Statistical Analysis

Analysis of results was performed grouping by time point (0, 14 and 21 days of CTX and during OM episode). The group of patients who developed oral mucositis at any stage of chemotherapy was compared with the group of patients who never developed it.

The Shapiro–Wilk normality test was performed, and based on the distribution of the data, parametric and non-parametric tests were chosen. ANOVA and Kruskal–Wallis rank sum tests were applied to analyze the statistical differences between groups by time point. Student’s *t* test and the Mann–Whitney U test were applied to analyze differences between groups according to the development of OM.

Spearman correlation coefficients were used to assess bacterial correlations with variables like saliva interleukins, OHI-S, laboratory values (e.g., neutrophils, lymphocytes, monocytes), among others. The correlation networks were generated in the software platform Cytoscape version 3.10.1. Significance value of *p* < 0.05.

## 3. Results

A total of 32 children between 2 and 16 years old (mean: 9 years; SD ± 4.8) were included in the study. Most of the children exhibited acceptable oral hygiene. The majority of the patients had cavities between grades 3 and 6; the cariogenic risk for almost all of them fell within the low to moderate range. The detailed clinical and oral characteristics of all children are listed in Table 1.

Oral rinse samples were collected from all 32 patients during the induction phase at three time points (days 0, 14, and 21), totaling 90 samples. Only four patients developed OM during the induction phase (between day 0 and day 21 of CTX); however, it was not possible to obtain a sample from one of them during the mucositis episode. Upon conducting follow-up (medical record), it was found that out of the 32 patients, 13 (40.6%) developed OM during the following phases of CTX; however, no samples were collected in those phases. An analysis of the age (Mann–Whitney U test, *p* = 0.42) and gender (chi-square test, *p* = 0.47) characteristics in both groups revealed no statistically significant differences (Table 2).

### 3.1. Alpha and Beta Diversity on Days 0, 14 and 21 of CTX and in Patients Who Did or Did Not Develop Oral Mucositis

From 32 children, a total of 90 oral rinse samples were collected on days 0, 14 and 21 of CTX. In total 9,641,089 qualified sequence reads were obtained and used for analysis, with an average of 104,794 sequence reads for each sample. Two samples had low sequence counts and were eliminated. The α diversity was evaluated using the Shannon and Chao1 indices.

**Figure 2:**
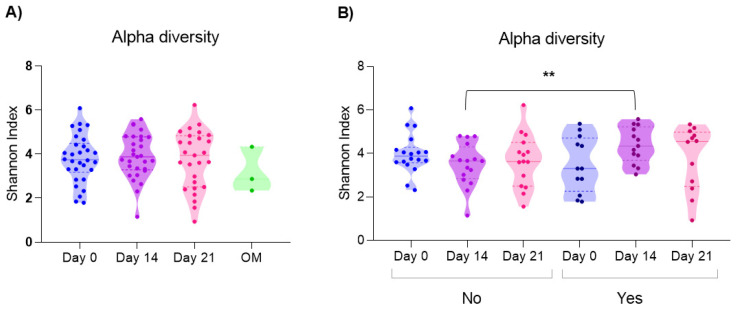
Alpha diversity based on the Shannon Index (**A**) by time point: on days 0 (*n* = 30), 14 (*n* = 29) and 21 (*n* = 27) of chemotherapy, and during oral mucositis, where no statistically significant difference was identified (ANOVA *p* = 0.66). (**B**) When comparing the alpha diversity of the groups according to whether they developed oral mucositis, it was observed that the Shannon index on day 14 was significantly higher in patients who did develop oral mucositis (ANOVA No *p* = 0.60; Yes *p* = 0.19. *t* test: day 0 *p* = 0.24; day 14 ** *p* = 0.01; day 21 *p* = 0.81) (No: day 0 *n* = 18, day 14 *n* = 16, day 21 *n* = 15; Yes: day 0 *n* = 12, day 14 *n* = 13, day 21 = 14). OM: during oral mucositis; No: patients who did not develop oral mucositis; Yes: patients who developed oral mucositis. The colors represent the different time points: Day 0 (blue), Day 14 (purple), Day 21 (pink), and samples taken during active mucositis (green).

It was observed that the Shannon index increased from day 0 (mean: 3.79 ± 1.0) to day 14 (mean: 3.89 ± 1.0) and subsequently, on day 21 (mean: 3.73 ± 1.0), they returned to a level similar to that observed on day 0. In addition, less diversity was identified at the time of OM (2.87) (*n* = 3). However, no statistically significant difference was identified in α diversity between groups (*p* = 0.75) (Figure 2A).

Interestingly, when comparing the α diversity of patients who did not develop OM with respect to those who did develop OM, it was observed that the group of children who did develop OM at some point during CTX had greater α diversity on day 14 (mean: 4.39 ± 0.8) compared to children who did not develop OM (mean: 3.4 ± 0.9) (*p* = 0.01) (Figure 2B).

Principal coordinate analysis (PCoA) based on the weighted UniFrac metric was also performed. Structural comparisons of oral microbiota between groups did not show differences in β diversity (Figure S1 of the Supplementary Material).

### 3.2. Relative Abundance on Days 0, 14 and 21 of CTX and in Patients Who Did or Did Not Develop Oral Mucositis

**Figure 3:**
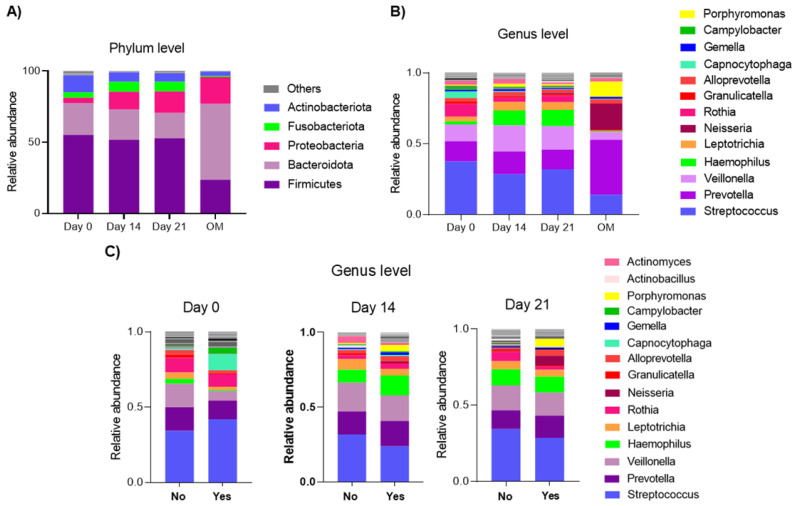
Relative abundance (**A**) at the phylum level, where it is observed that the relative abundances are similar between days 0, 14 and 21 of chemotherapy, with a predominance of *Firmicutes*. On the other hand, at the time of oral mucositis, the phylum *Bacteroidota* predominated. (**B**) At the genus level by time point (days 0, 14 and 21 of chemotherapy), important variations are also observed, especially during oral mucositis. (**C**) The relative abundance at the genus level is shown in patients who developed oral mucositis and those who did not develop it (based on the medical records) and by time point (days 0, 14 and 21 of chemotherapy). Note that the RA of microorganisms in patients who did not develop mucositis is more significantly altered on day 14 of chemotherapy. In the case of patients who developed mucositis, RA varies with the days of chemotherapy. By day 21, the RA of microorganisms in patients who did and did not develop oral mucositis was somewhat similar, compared with days 0 and 14. OM: during oral mucositis; No: patients who did not develop oral mucositis; Yes: patients who developed oral mucositis.

The comparison of the oral microbiota on days 0, 14, 21 and during the episode of OM was carried out at different taxonomic levels. A total of 12 phyla were identified, among those with the highest RA were *Firmicutes*, *Bacteroidetes*, *Actinobacteria*, *Proteobacteria* and *Fusobacteria* on days 0, 14 and 21. Some phyla appear to decline in RA while others increase. The phyla *Firmicutes* and *Bacteroidota* appear to remain stable during CTX. On the other hand, the phyla *Proteobacteriota* and *Fusobacteriota* increased their RA, while the *Actinobacteriota* phylum decreased during CTX and at the time of mucositis. During OM, the absence of the *Fusobacteriota* phylum was identified, as well as a notable decrease in the RA of the *Firmicutes* phylum, while the predominant phylum during the OM was *Bacteroidota* (Figure 3A).

At the genus level, *Streptococcus*, *Prevotella*, *Veillonella*, *Haemophilus* and *Leptotrichia* predominated. However, they present variations. From day 0 to days 14 and 21, *Capnocytophaga* is more abundant on day 0, and decreases during the treatment, while an increase in the RA of *Haemophilus* and *Leptotrichia* was observed. In the group at the time of OM, the RA of the genus *Prevotella*, *Neisseria* and *Porhyromonas* is notable, and the decrease in the RA of *Streptococcus*, *Veillonella* and *Haemophilus* (Figure 3B).

In patients who did not develop mucositis, lower RA of *Rothia* was identified as the days of CTX passed. On the other hand, there was a higher RA of *Actinomyces* on day 14 compared to patients who did develop OM. It was observed that the RA of *Streptococcus* appears to remain more stable in the group of patients who did not develop OM.

Patients who developed OM had higher RA of *Capnocytophaga* on day 0. In this group of patients, higher RA of *Neisseria* and *Porphyromonas* was also identified on days 14 and 21. On day 14, a greater variation was observed when comparing the RA of patients who did develop OM with those who did not, while on day 21, the RA of both groups showed greater similarity (Figure 3C).

### 3.3. Differential Abundance on Days 0, 14 and 21 of CTX and in Patients Who Did or Did Not Develop Oral Mucositis

**Figure 4:**
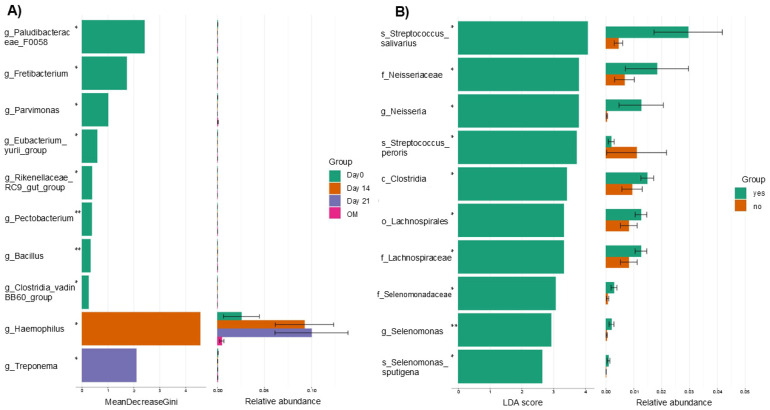
Differential abundance analysis. (**A**) Representative microorganisms of each group are shown by time point (day 0, 14, 21, and during oral mucositis). On day 0, the genera *Pectobacterium* and *Bacillus* can be mentioned (Kruskal–Wallis: * *p* < 0.05; ** *p* < 0.01). On day 14, *Haemophilus* was found to be the most representative genus, and on day 21, it was *Treponema*. (**B**) The same analysis was performed on day 14 in patients who did or did not develop oral mucositis based on their records, and it was found that *Selenomonas* and *Streptococcus*, among others, were the representative microorganisms of the group of patients who did develop oral mucositis (Kruskal–Wallis: * *p* < 0.05. ** *p* < 0.01). OM: during oral mucositis; No: patients who did not develop oral mucositis; Yes: patients who developed oral mucositis.

In the analysis of differential abundance (Random Forest) by time point, it was identified that the microorganisms that contributed to the differences between groups were: day 0: *F0058* (*Paludibacteraceae*), *Fretibacterium*, *Parvimonas*, *Eubacterium yurii*, *Rikenellaceae RC9 gut group*, *Pectobacterium*, *Bacillus* and *Clostridia vadinBB60 group*; day 14: *Haemophilus*; day 21: *Treponema* (Kruskal–Wallis: *p* < 0.05) (Figure 4A). A statistically significant difference (KW *p* = 0.03) was observed in the genus *Haemophilus,* which was representative on day 14 of CTX. In addition, we observed that the abundance of *Haemophilus* increased over the course of CTX. However, its levels decreased in patients at the time of OM onset (Figure 4A).

When comparing patients who did or did not develop OM, on day 14, representative taxa of the group that did develop OM were identified: *Streptococcus salivarius*, *Neisseria*, *Streptococcus peroris*, *Selenomonas sputigena*, *Clostridia*, and *Lachnospirales*. Only *Streptococcus peroris* was more abundant in the group of patients who did not develop mucositis (Figure 4B).

### 3.4. Cytokine Profile in Saliva in Patients with ALL on Days 0, 14 and 21 of CTX and in Patients Who Experienced OM During Subsequent Phases of CTX vs. Children Who Never Developed OM

**Figure 5:**
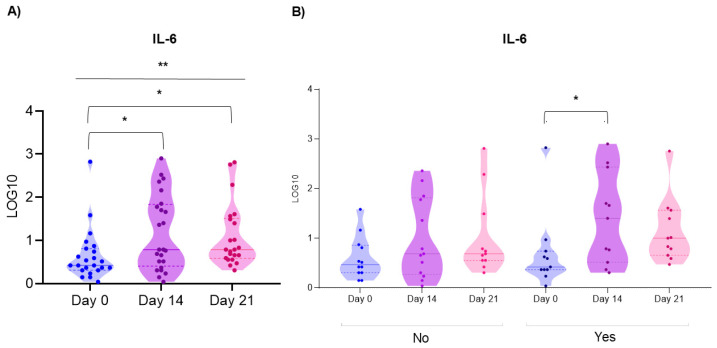
IL-6 concentration in saliva. (**A**) by time point (days 0, 14, and 21 of chemotherapy and during oral mucositis), where a statistically significant increase in the concentration of this interleukin was identified as the days of chemotherapy increased (Kruskal–Wallis; * *p* < 0.05; ** *p* < 0.01). (**B**) when comparing according to the development or not of oral mucositis, it was identified that patients who did develop oral mucositis presented a statistically significant increase in IL-6 from day 0 to day 14 of chemotherapy (Kruskal–Wallis * *p* = 0.03). No: patients who did not develop oral mucositis; Yes: patients who developed oral mucositis. Data normalized to a logarithmic scale of 10. The colors represent the different time points: Day 0 (blue), Day 14 (purple), Day 21 (pink).

A total of 80 non-stimulated saliva samples were collected. Overall, it was observed that the concentration of IL-6, IL-1β, IL-17 and MMP1 increased from day 0 to day 14, and decreased on day 21, although they did not reach the initial concentration (day 0). Interleukins IL-4 and IL-8 increased on days 14 and 21. TNF-α increased by day 14 and decreased beyond baseline by day 21. Statistically significant differences (*p* = 0.001) were only observed in the concentration of IL-6 in saliva. This interleukin increased notably from day 0 (2.49 pg) to day 14 (6.15 pg) and from day 0 to day 21 (5.83 pg) (Figure 5A).

Higher concentrations of all cytokines were found in the group of those who did develop OM vs. those who did not develop it (No). A statistically significant difference (*p* = 0.03) was only observed in the concentration of IL-6 in the group of those who developed OM, with an increase from day 0 (0.68 pg) to day 14 (1.39 pg) (Figure 5B).

### 3.5. Correlations in Patients Who Did or Did Not Develop Oral Mucositis

**Figure 6:**
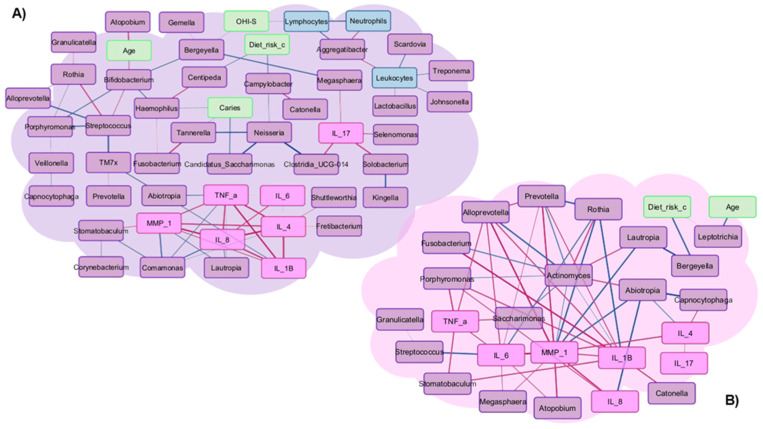
Correlation networks (day 14 of CTX) between clinical variables (green), laboratory variables (blue), salivary cytokines (pink), and bacteria (purple). The Pearson correlation coefficient shows whether there is a positive correlation (red line) or a negative correlation (blue line) between variables. A *p*-value less than or equal to 0.05 was considered significant, and r = 0.4 was the cutoff. (**A**) in patients who developed mucositis and (**B**) in patients who did not develop oral mucositis.

The correlation networks of the oral microbiome with some salivary cytokines, oral clinical variables and laboratory tests of patients who developed OM or not are shown in Figure 6. These correlation networks show the relationship between microorganisms and cytokines; however, it is not clear how these bacteria relate to clinical and laboratory variables in the study groups.

In the group of patients who did develop mucositis, a positive correlation between interleukins was observed, as expected. As mentioned above, there was a significant increase in IL-6 concentrations in this group of patients, indicating that other cytokines that were positively correlated also increased (Figure 6A).

*Selenomonas*, a pathogenic bacterium representative of the patient group who developed mucositis, was positively correlated with IL-17 (r = 0.67, *p* = 0.018). On the other hand, *Streptococcus*, a genus also representative of this patient group, was positively correlated with bacteria that are part of the normal oral microbiota such as *Rothia* (r = 0.73, *p* = 0.008) and negatively correlated with pathogenic bacteria such as *Porphyromonas* and *Alloprevotella* (r = −0.72, *p* = 0.01 and r = −0.74, *p* = 0.007, respectively). This seems to indicate that *Streptococcus* plays an important role in oral health, although it had a lower RA in this patient group.

In the group of patients who did not develop mucositis, it was observed that *Actinomyces*, a genus with a notable RA in these patients, was negatively correlated with inflammatory cytokines such as IL-1β (r = −0.62, *p* = 0.028) and MMP1 (r = −0.59, *p* = 0.037), as well as with pathogenic bacteria such as *Porphyromonas* (r = −0.55, *p* = 0.026), *Fusobacterium* (r = −0.62, *p* = 0.012), *Alloprevotella* (r = −0.74, *p* = 0.002) and *Prevotella* (r = −0.62, *p* = 0.011). On the contrary, a positive correlation was observed between *Actinomyces* and *Rothia* (r = 0.69, *p* = 0.004), *Lautropia* (r = 0.65, *p* = 0.008) and *Abiotropia* (r = 0.66, *p* = 0.007). This suggests that patients who did not develop mucositis had a higher abundance of non-pathogenic bacteria and therefore less inflammation (Figure 6B).

## 4. Discussion

To our knowledge, this is the first study conducted in pediatric patients with ALL in which the oral microbiome was assessed before and during CTX in patients who did and did not develop OM. We observed that the most significant changes in the oral microbiome occurred on day 14 of CTX, especially in patients who developed OM, in whom greater α diversity was identified, as well as an increase in the relative and differential abundance of mainly opportunistic microorganisms. Higher IL-6 concentrations were also observed in this group, and in all patients over the course of CTX. Furthermore, RA in patients who did not develop mucositis remained more stable than that of those who did. This may be a sign of dysbiosis and an early marker for the development of OM.

The oral microbiome is a determining element in maintaining homeostasis in the oral cavity; however, various factors can disrupt it [3,6,15]. Changes in the microbiome are characterized by an alteration in the diversity, abundance and composition of the oral microbiota [16]. Through sequencing, it has been shown that in patients with ALL, there is an imbalance of the microbiome before starting CTX, caused directly by the neoplasia [17]. Likewise, there is evidence that indicates that during CTX, the oral microbiome experiences dysbiosis [18]. We observed that patients who developed mucositis at some point during treatment had greater α diversity compared to those who did not develop mucositis. On the other hand, lower α diversity was observed at the time of mucositis. The interpretation of α diversity as an indicator of health or disease can be context-specific, although in some areas of the body, such as the gut, higher α diversity is an indicator of health, it has been shown that in the oral cavity any variation, whether higher or lower α diversity, is related to local and systemic pathological processes, such as severe periodontal disease, caries, obesity, and cancer, among others [19].

Ye et al., conducted a prospective study on the dynamics of the oral microbiome in pediatric patients with some type of neoplasia who underwent CTX. Similar to our study, they found that patients who developed mucositis had a more diverse bacterial community from the start of CTX [20]. In another study conducted by Hong, dysbiosis was shown to significantly contribute to epithelial damage and, consequently, to the development of mucositis, with significant changes in the epithelial transcriptome, as well as upregulation of genes related to the innate immune response and apoptosis [3].

The most significant changes in the microbiota composition were observed on day 14 of chemotherapy, coinciding with the induction phase. At this point in the treatment for ALL, high-dose medications are used to reduce tumor cells and achieve disease remission [21]. The alteration of the microbiota during this phase, induced by chemotherapy, is a key factor that predisposes patients to the development of oral mucositis (OM). Critically, these differences in microbiome composition are observed before the clinical onset of OM, suggesting that specific microbial profiles may serve as early indicators for the development of this condition. The drugs most frequently associated with this effect, such as alkylating agents, anthracyclines, and antimetabolites, repress gene transcription and replication, inhibiting DNA synthesis and blocking mitosis [5,8]. This cytotoxic effect significantly alters the interaction between keratinocytes, the immune system, and the oral microbiome, which favors the development of OM [6,8,22]. Although chemotherapeutics have antimicrobial activity, they reduce the number of neutrophils, resulting in local immunosuppression and an increased bacterial load [3]. This environment promotes the growth of anaerobic Gram-negative microorganisms, which release lipopolysaccharides (LPS) that stimulate macrophages to produce inflammatory molecules such as IL-6, IL-1, and TNF, thereby aggravating the mucosal lesion [23].

An important finding was that patients who did not develop mucositis showed a more stable RA of the main genera throughout chemotherapy, in contrast to those who developed mucositis, who exhibited greater fluctuations. Additionally, we found that on day 21 of CTX, the AR in patients who did develop mucositis and those who did not develop it were more similar to each other, compared to days 0 and 14 of CTX. This suggests that despite experiencing alterations in their oral microbiome during treatment, patients with OM have the capacity to revert to a baseline-like state. Resilience is defined as the ability to resist change and persist, while stability is defined as the ability to return to a state of equilibrium after a temporary disturbance [24]; both can be related to health conditions. One of the most temporally stable microbiomes in the human body is the oral microbiome [25]. However, its instability or dysbiosis can lead to complications such as mucositis [20,26]. Our results are consistent with Ye et al., who identified that patients who developed OM had more pronounced changes in bacterial composition after the start of CTX, while patients who did not develop mucositis maintained a more stable β diversity before and during CTX, meaning that their microbiome was apparently less affected during CTX [20].

In a study based on the V1–V3 variable region in patients with ALL, no significant differences were found in gut microbiome diversity from diagnosis to end of maintenance; however, they report that gut microbiome diversity decreased significantly after induction and reinduction, a period where CTX is intensified. This suggests that the diversity can recover, although the composition changes [4]. Similarly, another study conducted in patients with leukemia found that the mean colony-forming units of *S*. *mutans* pre- and post-treatment with CTX were similar, although lower than in healthy subjects. This study suggests that changes in these values are more related to treatment than to the disease; therefore, once treatment with CTX is completed, patients could return to their baseline state, depending on how stable their oral microbiome is [27].

Changes in the abundance and composition of the microbiome may result from a shift in microorganisms from a commensal state to the growth of pathobionts [3,6]. In the present study, it was observed that from day 0 of CTX, all patients presented dysbiosis, because the bacteria representative of the different days of treatment are mostly anaerobic microorganisms belonging to the red complex, such as *Parvimonas*, *Eubacterium yurii*, *Fretibacterium* and *Treponema*, considered indicators of periodontal and pulp disease [3,16,28]. These bacteria have also been observed in subjects who smoke, with candidiasis, oral or distant cancer, or have OM [2,22,29,30,31]. Although the genera *Haemophilus* and *Treponema* are part of the oral microbiota of adults, they include non-pathogenic and pathogenic species, which can cause a wide variety of infections in infants and children [32].

The phylum *Firmicutes*, currently named *Bacillota*, comprises more than 250 genera, including *Bacillus*, *Clostridium*, and *Streptococcus*, and is one of the most predominant phyla in the soft tissues of the oral cavity and in saliva [33], particularly in children with ALL [17,34]. Members of this phylum can be either beneficial or harmful depending on the context; most are acid producers and participate in fermentation pathways [35]. Within this phylum, *Streptococcus* is the predominant genus in the saliva of healthy children [36]. These bacteria include potent symbionts capable of producing molecules that inhibit pathogenic microorganisms; however, their abundance has been reported to decrease during chemotherapy [3,37]. Most oral *Streptococcus* species regulate inflammatory pathways in host cells and are associated with oral health [31,38]. On the other hand, our study identified *S*. *salivarius*, a Gram-positive commensal that predominates in saliva [16,28], as a representative microorganism in patients who subsequently developed mucositis. Although typically harmless, *S*. *salivarius* can shift to a pathogenic phenotype under certain conditions, such as immunosuppression or CTX, leading to local or systemic infections [36,37]. This genus can produce enzymes that degrade mucin and produce a detrimental effect on the oral mucosa [37]. Similarly, in the study by Ye et al., it was reported that in patients who later developed mucositis, the *Streptococcus* genus increased, although they did not mention the species [20].

Other representative genera of the group of patients who developed mucositis were *Neisseria* and *Selenomonas*. The *Neisseria* genus is the most predominant member of the *Proteobacteria* phylum. These Gram-negative cocci are commonly part of the normal oropharyngeal microbiota and are integral components of the oral core microbiome [29,39]. However, an increase in the abundance of this genus has been reported in immunosuppressed patients, indicating that some species are opportunistic pathogens [30]. Some species are highly autolytic and release peptidoglycan fragments during growth, which are toxic to the mucosa and may contribute to the intensification of the inflammatory response during OM [40].

*Selenomonas* has been reported to appear exclusively after CTX and to increase its RA within 14 days of starting CTX [3]. These are Gram-negative anaerobic curved rods that are part of the normal gingival microbiota; however, their increased abundance is associated with periodontal disease and other inflammatory diseases outside the mouth, such as systemic lupus erythematosus [41]. Its pathogenic potential and virulence mechanisms are not clearly understood [42]. *S*. *sputigena* has been shown to contribute to inflammation and tissue destruction by binding to gingival keratinocytes and inducing the expression and secretion of pro-inflammatory cytokines and chemokines, such as IL-6, IL-17, and IL-33. It also influences the production of matrix metalloproteinases [16,41]. These properties may explain the positive correlation observed in our study between *Selenomonas* and inflammatory cytokine concentrations in patients who developed OM.

Interestingly, patients who developed mucositis had higher RA of *Capnocytophaga* compared to those who did not develop OM. This genus is part of the oral microbiota of children between 2 and 10 years of age, although its presence has been reported in patients receiving CTX [43,44]. It has been reported that there may be an overgrowth of these bacteria in immunocompromised patients, especially in patients with hematological malignancies [43,44,45]. In addition, some of its species have been found in between 50 and 80% of patients with OM [45].

In our study, approximately half of the patients developed OM at some point during CTX; however, only four experienced mucositis within the first 21 days of treatment. The overall prevalence of OM is estimated at around 45.1%, though this rate varies depending on factors such as the type of neoplasia, treatment modality, patient population, and age, among others [6,8,46]. Most studies report that patients usually experience this adverse effect after 7–14 days after the start of CTX [8]. The patients included in this study developed mucositis in advanced stages of CTX, probably because several medications that have been related to the development of OM are still used in maintenance, although at lower doses. Patients with ALL receiving CTX who develop mucositis show short- and long-term microbiome changes like a decrease in commensal bacteria and an increase in microorganisms related to inflammatory processes [3,18,26].

During a mucositis episode, a notable increase in the RA of the *Bacteroidota* phylum was identified. In addition to being present in healthy patients with mixed and permanent dentition [34], it is also among the most predominant in children with leukemia [18] and in patients who have undergone CTX [3]. It includes Gram-negative anaerobic genera such as *Prevotella* and *Porphyromonas*, usually related to periodontal disease and other inflammatory and ulcerative processes of the oral cavity, such as lichen planus, recurrent aphthous stomatitis, and mucositis [28,47,48].

An increase in IL-6 concentration in saliva was identified overall, particularly on day 14 of CTX, in patients who developed OM at any phase of treatment. This interleukin plays an important role in the cytotoxic damage caused by CTX, as well as in the development of OM, according to the mucositis pathogenesis model proposed by Sonis [5]. Although OM is not an infectious disease, bacterial colonization following its development can prolong healing time. Colonization of the ulcerated surface stimulates macrophages, contributing to the production of pro-inflammatory cytokines such as IL-6, suggesting a predominance of inflammatory processes in the initial stages of treatment [2,5,8,21]. These findings are consistent with the study by Morales et al., conducted in pediatric patients with leukemia, which reported higher concentrations of interleukins, including IL-6, in saliva during CTX treatment [49].

Among the bacteria associated with OM are Gram-negative microorganisms such as *P*. *gingivalis* and *P*. *intermedia*. These species contain lipopolysaccharide (LPS) endotoxins in their cell walls, which activate macrophages to produce and upregulate cytokines and inflammatory mediators, including IL-1, IL-6, matrix metalloproteinases, and TNF-α, thereby aggravating mucosal inflammation [31,50]. *P*. *gingivalis* LPS also disrupts the epithelial barrier by breaking down cell–cell and cell–extracellular matrix junctions, including claudin, occludin, E-cadherin, and catenins, through soluble virulence factors such as gingipains, which are highly active proteases [51,52]. Moreover, *P*. *gingivalis* can degrade cell signaling molecules and inactivate functions essential for wound healing, such as epithelial cell migration, by altering keratinocyte adhesion to laminin-5, a key extracellular matrix component [52]. Its outer membrane vesicles further inhibit angiogenesis and reduce endothelial cell and fibroblast proliferation, delaying healing, while also activating pathogen recognition receptors to induce cytokine production and apoptosis [53,54]. Additionally, *P*. *gingivalis* has been associated with pro-inflammatory mechanisms, including induction of cyclooxygenase-2 (COX-2) expression and prostaglandin E2 production via ERK1/2 kinase activation in monocytes [31,55].

Similarly, *Prevotella* species, which are common oral commensals from early life, can become opportunistic pathogens in susceptible hosts, with increases in their abundance linked to dysbiosis [50,56]. *Prevotella* has been reported to induce epithelial cells to produce antimicrobial peptides and activate cytokines such as IL-1, IL-8, and IL-17, as well as signaling pathways like NF-κB [56]. The activation of these inflammatory pathways, including IL-6 upregulation, positions *Prevotella* as an indicator of early and severe OM development [3,31]. Together, these findings highlight the role of Gram-negative bacteria such as *P*. *gingivalis* and *Prevotella* in promoting inflammation through the induction of cytokines like IL-6, thereby contributing to the pathogenesis and progression of OM during CTX. Moreover, excipients in oral formulations, such as fast-dissolving lyophilizates, can influence drug dissolution, stability, and local tolerance, potentially modulating the microbiome and mucosal immune responses [57].

Within the limitations of this study are the few cases of oral mucositis during the induction phase, as well as the reliance on clinical diagnoses from medical records for oral mucositis grading without a pre-specified inter-examiner calibration protocol, which restricted sampling during acute episodes and prevented definitive group comparisons, focusing instead on temporal microbial dynamics within individuals. Additionally, the absence of prior pediatric mucositis studies reporting Shannon index variability limited formal power calculations, and the single-center design may reduce generalizability. Inter-individual variability in microbiota composition could also influence associations with disease.

This study represents the first characterization of oral microbiota dynamics in pediatric patients with acute lymphoblastic leukemia (ALL) during chemotherapy. The findings open the possibility of applying preventive strategies based on microbiome monitoring. Understanding how the oral microbiota changes before mucositis onset may contribute to the development of interventions aimed at modulating the microbiota to reduce the risk or severity of mucositis.

## 5. Conclusions

Children with ALL undergoing chemotherapy show changes in the diversity and relative abundance of the oral microbiome, with the greatest alterations observed on day 14. Microbial dysbiosis may predict CTX-related complications; however, further studies are needed, particularly in the pediatric oral microbiome, due to the limited existing literature.

The detection of inflammatory mediators in saliva provides a local, non-invasive overview of inflammation, allowing identification of changes that occur before the ulcerative phase. One of the strengths of this study is that the samples were taken from patients who did not receive prophylactic or preventive treatment for OM, and we were able to observe disease progression without medical intervention.

Finally, despite the limitations of this study, our findings provide a foundation for future research on microbial metabolomics and could inform conservative treatments or preventive strategies for complications in pediatric patients with ALL receiving CTX.

This study provides the first longitudinal characterization of oral microbiota dynamics in pediatric patients with acute lymphoblastic leukemia (ALL) during chemotherapy. The results support the hypothesis that specific microbial changes precede the onset of oral mucositis, suggesting that the oral microbiota may serve as an early indicator of mucosal toxicity. These findings contribute to a better understanding of host–microbiome interactions during chemotherapy and open up the possibility of developing preventive strategies through microbiota modulation to reduce the risk of mucositis in pediatric oncology patients.

## Figures and Tables

**Table 1:** Demographic data, oral hygiene and caries index from 32 ALL patients.

Variable		*n*	Percentage (%)
Gender			
Female		16	50
Male		16	50
Total		32	100
Age (years)			
0–5		11	34.4
6–13		12	37.5
+14		9	28.1
Total		32	100
OHI-S			
Poor		1	3.2
Fair		17	53.1
Good		12	37.5
Lost		2	6.2
Total		32	100
Dental caries			
0		4	12.5
1–2		4	12.5
3		8	25
4–5		7	21.9
6		7	21.9
Lost		2	6.2
Total		32	100
Cariogenic risk of diet			
Low		14	43.7
Moderate		14	43.7
High		2	6.2
Lost		2	6.2
Total		32	100

OHI-S: Oral Hygiene Index Simplified. Caries 0: no evidence of caries; 1: change in enamel opacity; 2: change in enamel when wet; 3: enamel breakdown; 4: underlying dark shadow from dentine; 5: cavity with visible dentine; 6: extensive, distinct cavity with visible dentine.

**Table 2:** Characteristics of ALL patients who developed OM.

Variable		*n*	Percentage (%)
Gender			
Female		5	38.5
Male		8	61.5
Total		13	100
Median age (years)		9	
Age (years)			
0–5		5	38.5
6–13		5	38.5
+14		3	23
Total		13	100
Development of OM			
Induction		4	30.7
Consolidation		7	53.8
Maintenance		2	15.4
Total		13	100
Grade of OM			
I		1	7.7
II		3	23
III		1	7.7
IV		3	23
Not specified		5	38.5
Total		13	100

Grade according on the World Health Organization OM classification. ALL: Acute lymphoblastic leukemia; OM: Oral mucositis.

## Data Availability

The original data presented in the study are openly available in the Sequence Read Archive (SRA) repository under the Bioproject number PRJNA1329414.

## Supplementary Materials

The following supporting information can be downloaded at https://www.mdpi.com/article/10.3390/microorganisms14010185/s1. Figure S1: Weighted Unifrac PCoA analysis. No statistical differences were observed between groups by time point or development of oral mucositis.

## Abbreviations

The following abbreviations are used in this manuscript:

ALL	Acute lymphoblastic leukemia
CTX	Chemotherapy
OM	Oral mucositis
IL	Interleukin
DNA	Deoxyribonucleic acid
TNF-α	Tumoral necrosis factor alpha
OHI-S	Oral hygiene index
ICDAS	International Caries Detection and Assessment System
PBS	Phosphate-buffered saline
MMP	Matrix metalloproteinases
RNA	Ribonucleic acid
PCR	Polymerase chain reaction
PCoA	Principal coordinate analysis
RA	Relative abundance
KW	Kruskal–Wallis
COX-2	Cyclooxygenase-2
