# Supplementary figures and images for: Oral Microbiome Dynamics in Patients with Acute Lymphoblastic Leukemia and Oral Mucositis
Source: Microorganisms. 2026 Jan 14;14(1):185. doi: 10.3390/microorganisms14010185 (PMC12843840; doi:10.3390/microorganisms14010185)

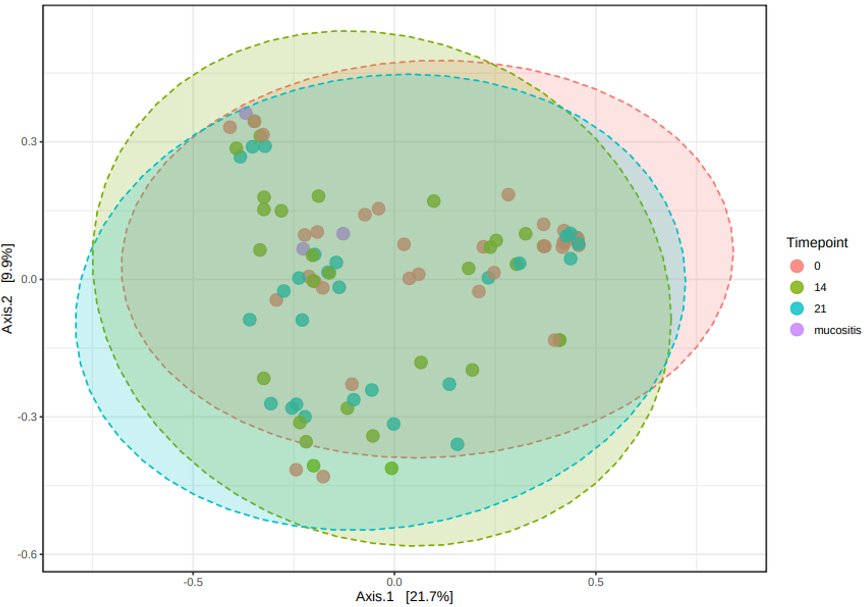

Supplement: Supplementary file 1 [file microorganisms-14-00185-s001.zip › microorganisms-3908809-supplementary.tif]
